# Local and Systemic Effect of Cytokinins on Soybean Nodulation and Regulation of Their *Isopentenyl Transferase* (*IPT)* Biosynthesis Genes Following Rhizobia Inoculation

**DOI:** 10.3389/fpls.2018.01150

**Published:** 2018-08-08

**Authors:** Celine Mens, Dongxue Li, Laura E. Haaima, Peter M. Gresshoff, Brett J. Ferguson

**Affiliations:** Centre for Integrative Legume Research, School of Agriculture and Food Sciences, The University of Queensland, Brisbane, QLD, Australia

**Keywords:** autoregulation of nodulation, cytokinin, legumes, nodulation, plant signaling and development, symbiosis, IPT, isopentenyltransferase

## Abstract

Cytokinins are important regulators of cell proliferation and differentiation in plant development. Here, a role for this phytohormone group in soybean nodulation is shown through the exogenous application of cytokinins (6-benzylaminopurine, N^6^-(Δ^2^-isopentenyl)-adenine and *trans*-zeatin) via either root drenching or a petiole feeding technique. Overall, nodule numbers were reduced by treatment with high cytokinin concentrations, but increased with lower concentrations. This was especially evident when feeding the solutions directly into the vasculature via petiole feeding. These findings highlight the importance of cytokinin in nodule development. To further investigate the role of cytokinin in controlling nodule numbers, the *IPT* gene family involved in cytokinin biosynthesis was characterized in soybean. Bioinformatic analyses identified 17 *IPT* genes in the soybean genome and homeologous duplicate gene partners were subsequently identified including *GmIPT5* and *GmIPT6*, the orthologs of *LjIPT3*. Expression of *GmIPT5* was upregulated in the shoot in response to nodulation, but this was independent of a functional copy of the autoregulation of nodulation (AON) receptor, GmNARK, which suggests it is unlikely to have a role in the negative feedback system called AON. Legumes also control nodule numbers in the presence of soil nitrogen through nitrate-dependent regulation of nodulation, a locally acting pathway in soybean. Upon nitrate treatment to the root, the tandem duplicates *GmIPT3* and *GmIPT15* were upregulated in expression indicating a role for these genes in the plant’s response to soil nitrogen, potentially including the nitrate-dependent regulation of legume nodulation pathway. Additional roles for cytokinin and their *IPT* biosynthetic genes in nodulation and the control of nodule numbers are discussed.

## Introduction

Legumes are economically important food, feed and fuel crops (Gresshoff et al., 2015). The majority are able to engage in a symbiotic relationship with nitrogen-fixing soil bacteria referred to as rhizobia, allowing legumes to grow under low soil nitrogen conditions. While rhizobia fix atmospheric nitrogen (N_2_) into ammonia that is readily assimilated by the plant, rhizobia in return receive photosynthetic carbohydrates in the protected nodule environment.

Reciprocal plant-microbe signaling is required to establish this relationship, which results in the formation of new root organs called nodules. Both nodulation and nitrogen fixation come at a high energy and resource cost to the plant. It is therefore important for the host to regulate its nodule numbers. This can be achieved via a nitrate-dependent regulation of nodulation mechanism in the presence of high soil nitrogen and through autoregulation of nodulation (AON) (Ferguson et al., 2010; Reid et al., 2011; Ferguson et al., 2018). The AON pathway is a negative feedback system involving extensive signaling between the root and shoot. Following rhizobia inoculation, plant-derived CLAVATA3/embryo-surrounding region (CLE) peptide signals are produced in the root (Reid et al., 2011; Hastwell et al., 2015a,b, 2018). These peptides are transported via the xylem to the shoot where they are perceived by a receptor complex that centers around a CLAVATA1 (CLV1)-like leucine-rich repeat receptor kinase [such as GmNARK in *Glycine max* (soybean), LjHAR1 in *Lotus japonicus* and MtSUNN in *Medicago truncatula*] (Okamoto et al., 2009, 2013; Mortier et al., 2010; Reid et al., 2011). Perception of the CLE peptides results in the differential regulation of a novel shoot-derived signal that travels back down to the roots to regulate nodule numbers (Ferguson et al., 2010; Ferguson et al., 2018). The shoot-derived signal and many other factors acting in the control of nodule numbers, are currently unknown or poorly understood.

Phytohormones are important signals involved in the formation and regulation of legume nodules (reviewed by Ferguson and Mathesius, 2014). Cytokinin hormones are a class of structurally similar N^6^-substituted adenine derivatives with a central role in plant growth and development. Since their discovery in cell proliferation and differentiation, several other functions have been attributed to this class of phytohormones, including maintenance of the shoot apical meristem, branching, organogenesis, delay of senescence, long-distance communication of nutritional status and the plant’s response to biotrophic pathogens (reviewed by Kamada-Nobusada and Sakakibara, 2009). Like auxins, they can act both locally and as long-distance messengers. Natural cytokinins are classified into aromatic and isoprenoid cytokinins, the latter are most prevalent in higher plants and include *trans*-zeatin (tZ), *cis*-zeatin (cZ), dihydrozeatin (DZ) and N^6^-(Δ^2^-isopentenyl)-adenine (2-iP). Cytokinins show further variation through the addition of side chains, which seem to confer receptor specificity. Kinetin and 6-benzylaminopurine (BAP) are synthetic cytokinins frequently used for research purposes.

In legumes, the exogenous application of cytokinins induces the formation of nodule-like structures through the induction of early nodulin genes and cortical cell divisions (Mathesius et al., 2000; Heckmann et al., 2011). Mutants defective in the cytokinin receptor histidine kinase, Cytokinin Response 1 (CRE1) in *M. truncatula* and its ortholog Lotus Histidine Kinase 1 (LHK1) in *L. japonicus*, lack the ability to effectively form nodule primordia, whereas gain-of-function mutants exhibit spontaneous nodule formation (Gonzalez-Rizzo et al., 2006; Murray et al., 2007; Tirichine et al., 2007; Plet et al., 2011). Moreover, *Lonely Guy* (*LOG*) genes encoding cytokinin riboside 5′-monophosphate phosphoribohydrolase, which are involved in the biological activation of cytokinins, were found to be expressed in the proliferating cells of the nodule primordium (Mortier et al., 2014). Upregulation of *LOG* genes resulted in less nodules and was independent of the leucine-rich repeat receptor kinase that acts in the shoot to perceive root-derived CLE peptide signals during AON (Mortier et al., 2014).

Isopentenyl transferases (IPTs) carry out the first and rate-limiting step in cytokinin biosynthesis, where an isopentenyl group is transferred to either AMP, ADP or ATP (Kakimoto, 2001). Recently, *IPT3* was shown to be required for nodule development in the model legume *L. japonicus* (Chen et al., 2014; Sasaki et al., 2014; Reid et al., 2017). It has also been proposed that LjIPT3 could have a role in the synthesis of cytokinin molecules that act as the shoot-derived inhibitory factor in AON (Sasaki et al., 2014). LjIPT2 is thought to be responsible for the initial cytokinin build-up required for nodulation initiation, alongside LjLOG4 and independent of the LHK1 receptor (Reid et al., 2017). Interestingly, IPT encoding genes are also differentially expressed following treatment with certain nitrogen sources (Miyawaki et al., 2004), with nitrogen promoting plant development, but also inhibiting nodule organogenesis. However, it is important to remember that in addition to synthesis, post-transcriptional processing and degradation are also important to maintaining homeostasis of biologically active cytokinins.

Here, we report that exogenous application of low concentrations of cytokinin promotes nodule numbers in soybean, while high concentrations reduce them. This was achieved by treating soybean with either BAP, 2-iP or tZ type cytokinins using root-drench or petiole-feeding methods. These findings support the notion that cytokinin promotes nodule organogenesis, but suggest that the hormone is unlikely the shoot-derived factor of AON and that the inhibition we observe by feeding high concentrations is more likely the result of toxicity to the plant. In addition, 17 *IPT* genes were identified in the soybean genome and genetically characterized. Of these genes, the tandem duplicates *GmIPT3* and *GmIPT15* were upregulated in the root upon nitrate treatment, indicating a role for these genes in nitrate response. Upon rhizobia inoculation, *GmIPT5* (a soybean ortholog of *LjIPT3*) was significantly upregulated in the shoot following rhizobia inoculation of the root, consistent with previous reports (Chen et al., 2014; Sasaki et al., 2014). Upregulation of *GmIPT5* occurred in both the root and shoot in wild-type and GmNARK mutant plants (*nts382*), indicating that it functions in the leaf and root in response to nodulation, but likely does not have a role in the AON pathway.

## Materials and Methods

### General Plant and Bacterial Growth Conditions

Soybean lines used include wild-type Bragg and the GmNARK mutant lines *nts1116* (weak allele) and *nts382* (strong allele), a hypernodulating and supernodulating line, respectively (Carroll et al., 1985). Seeds were surface-sterilized overnight with chlorine gas or in 70% ethanol/3.5% H_2_O_2_ for 1 min followed by extensive rinsing with sterilized water.

Plants were grown in controlled glasshouse conditions with a 16 h/8 h photoperiod at 28 and 26°C, respectively and watered as required with a modified nutrient solution (Broughton and Dilworth, 1971; Herridge, 1982). For the nitrate treatments, the nutrient solution was supplemented with 10 mM of KNO_3_ in the week before harvest and applied every second day for 6 days prior to sample collection.

Plants were inoculated with *Bradyrhizobium diazoefficiens* CB1809/USDA110 (formerly *Bradyrhizobium japonicum*) or the incompatible Nod-factor mutant *nodC^−^*. The rhizobia were grown at 28°C in a yeast-mannitol broth for 3 days and inoculated at an optical density at 600 nm of 0.1. Nodule numbers were determined 9 days after inoculation and subsequently root dry weights were recorded.

### Cytokinin Treatments

Six-week old plants were treated with water as a control or three different types of cytokinin (BAP, 2-iP and tZ) at concentrations of 0.1 or 100 μM. The hormones were dissolved in MilliQ water and the treatments were administered through either root drenching (Lorteau et al., 2001) or petiole feeding (Lin et al., 2010, 2011). For the root-drench assay, the hormone solutions (150 ml) were poured directly onto the vermiculite every second day. For the petiole-feeding assay, a feeding apparatus consisting of a syringe and silicon tubing was constructed that enables the cytokinin solution to be fed continuously into the shoot via a severed petiole. This method directly supplies the solutions into the plant, including the phloem, which mimics the source of the shoot-derived inhibitor. In both cases, plants were inoculated with *B. diazoefficiens* CB1809 1 day after the start of feeding. Plants were harvested after 10 days of treatment.

### Bioinformatic Analysis

To identify the *IPT* gene family in soybean, common bean (*Phaseolus vulgaris*), *M. truncatula* and *L. japonicus*, multiple BLASTP searches were conducted against various online databases including Phytozome, NCBI, DOBLAST and Lotus Base using the LjIPT3 and the nine previously identified AtIPT amino acid sequences as a query (Takei et al., 2001; Miyawaki et al., 2004). Functional domains were predicted via the Pfam (EMBL-EBI) and PANTHER^TM^ v11.0 databases (Punta et al., 2012; Mi et al., 2017). Synteny between genomic environments was analyzed using Phytozome JBrowse. The obtained sequences were further characterized through a multiple sequence alignment via Clustal Omega hosted by EMBL-EBI (Goujon et al., 2010; Sievers et al., 2011; McWilliam et al., 2013; Corcilius et al., 2017). Phylogenetic trees were constructed using the PHYML plugin embedded in Geneious Pro v10.0.9 (Guindon and Gascuel, 2003) based on the protein sequence alignment adjusted by an algorithm in Geneious Pro v10.0.9 to retain only the homologous sequences and remove 75% of the gap regions. This plugin implements the maximum likelihood method to generate phylogenetic trees with 1,000 bootstraps supporting the branches.

### Gene Expression Analysis

For gene expression analyses, the first trifoliate leaf and the entire root from rhizobia-inoculated plants or root from 23-day-old nitrate-treated plants, were harvested, snap-frozen and homogenized in liquid nitrogen. The tissue was harvested 10 days after rhizobia inoculation of 2-week old plants. Total RNA was extracted the automated Maxwell^®^ LEV simplyRNA Tissue kit (Promega) according to the manufacturer’s protocol. The quality and quantity of the RNA samples were assessed using the NanoDrop^TM^ One Spectrophotometer (Thermo Fisher Scientific). SuperScript^®^ III Reverse Transcriptase (Invitrogen) was used to generate cDNA from 500 ng of DNase-treated RNA. An initial PCR on cDNA was done to identify *IPT* genes expressed in the different conditions. An RT-qPCR analysis was performed using the Roche LightCycler^®^ 96 with SYBR green fluorescence detection in a 96-well plate. All reactions were conducted in duplicate for at least two biological replicates and a target amplicon size of approximately 100 bp (**Supplementary Table S1**). The *GmCons6* housekeeping gene was included to normalize gene expression levels (Libault et al., 2008).

### Statistical Analysis

A statistical analysis was performed on all results using the GraphPad Prism 7 software. A Student’s *t*-test was used to determine the statistical significance of differences in nodule numbers assuming normal distribution of the data, root dry weights and relative expression of the target genes.

## Results

### Effect of Cytokinin Treatment on Soybean Nodulation

To determine the effect of cytokinin on soybean nodulation, 0.1 or 100 μM of BAP, tZ or 2-IP or a water control, were applied to the hypernodulating GmNARK mutant, *nts1116*. Concentrations lower than 0.1 μM were not applied as these are too low to induce a response in soybean. The hypernodulating mutant was used as a possible effect on nodule numbers caused by the cytokinin treatments would be easier to observe and quantify on a hypernodulating *nts1116* root system compared with the wild-type. The cytokinin treatments were administered using one of two methods; root-drenching or a petiole-feeding method to introduce the hormone to the shoot (Lin et al., 2010, 2011), after which the nodule number and root dry weight were determined.

**FIGURE 1:**
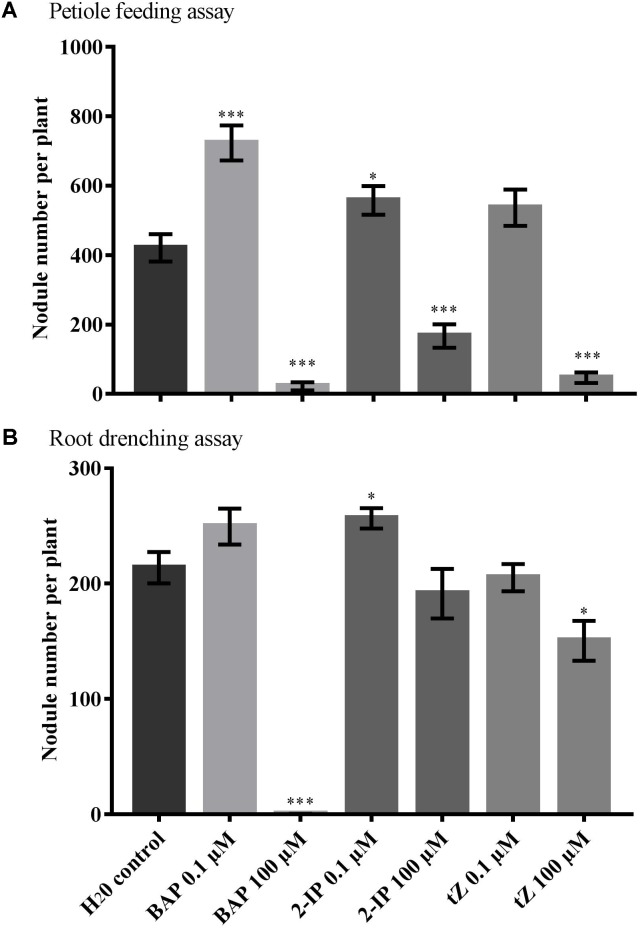
Effect of cytokinin treatment on nodule number. Supernodulating soybean plants (*nts1116*) were fed water (control) or cytokinin through **(A)** petiole feeding or **(B)** root drenching. Plants were inoculated with *Bradyrhizobium diazoefficiens* (CB1809) 1 day after the start of treatment. Nodule numbers were counted after 10 days of treatment (*n* = 8). Data and error bars represent mean ± SEM. Asterisks indicate significant differences in nodule number compared with the water control (Student’s *t*-test, ^∗∗∗^*P* ≤ 0.001, ^∗∗^*P* ≤ 0.01, ^∗^*P* ≤ 0.05).

Petiole feeding of 100 μM solutions with any of the three types of cytokinin significantly reduced nodule numbers compared with the water control (*P* < 0.001) (**Figure 1A**). In contrast, low 0.1 μM concentrations of both BAP and 2-IP significantly increase the number of nodules formed (*P* = 0.0004 and *P* = 0.030, respectively). A slight increase was also observed using 0.1 μM tZ, but this was not significant (*P* = 0.096). No significant differences in root dry weight were observed with any of the treatments used.

The root drenching results were similar, but generally less pronounced. Application of 100 μM BAP almost completely abolished nodulation (*P* < 0.0001), with 100 μM tZ also resulting in a significant reduction in nodule numbers (*P* = 0.012) (**Figure 1B**). Treatment with 100 μM 2-IP resulted in a slight, but not significant reduction (*P* = 0.390). Drenching with 0.1 μM of 2-IP significantly increased nodule numbers (*P* = 0.019), similar to what was observed using petiole feeding, whereas 0.1 μM of BAP or tZ led to a slight, but not significant, change in nodulation.

### Identification and Sequence Characterization of the *IPT* Gene Family in Soybean

To determine the role of cytokinin biosynthesis during the development and control of soybean nodulation, *IPT* gene expression was investigated. First, the complete *IPT* gene family was identified in soybean via multiple BLAST searches using the LjIPT3 amino acid sequence as a query. LjIPT3 was previously reported to have a role in nodulation and/or AON (Chen et al., 2014; Sasaki et al., 2014; Reid et al., 2017). The BLAST results lead to the identification of 17 *IPT* gene members in soybean having high sequence identity to LjIPT3 (**Table 1** and **Supplementary Table S2**). All of the translated IPT gene products were predicted via the Pfam database to possess a tRNA delta(2)-isopentenyl pyrophosphate (IPP) transferase domain, which is critical for the enzyme’s function in cytokinin biosynthesis, with the exception of GmIPT10, GmIPT15 and GmIPT17. However, additional searches using PANTHER^TM^ v11.0 confirmed the classification of GmIPT10, GmIPT15 and GmIPT17 within the IPP family of proteins. To further confirm their classification within the soybean *IPT* gene family and provide information on their potential function, synteny between their genomic environments was analyzed (see below, **Figure 3**).

**Table 1:** Features of the *IPT* gene family in soybean.

Name	Phytozome identifier	Chromosome location	Orientation	IPP transferase domain	Predicted introns	Protein length
GmIPT1	Glyma.10G273500.1	Chr10:49594484..49595398	Reverse	Y	0	304
GmIPT2	Glyma.20G116500.1	Chr20:35894331..35895981	Forward	Y	1	231
GmIPT3	Glyma.17G017400.1	Chr17:1313758..1315795	Forward	Y	0	340
GmIPT4	Glyma.07G256700.1	Chr07:43274336..43276366	Reverse	Y	0	336
GmIPT5	Glyma.10G025300.1	Chr10:2189157..2190507	Reverse	Y	0	344
GmIPT6	Glyma.02G148600.1	Chr02:15319273..15320731	Forward	Y	0	327
GmIPT7	Glyma.19G154400.1	Chr19:41471430..41472362	Reverse	Y	0	310
GmIPT8	Glyma.03G151800.1	Chr03:36688884..36690099	Reverse	Y	0	309
GmIPT9	Glyma.15G103800.1	Chr15:8102824..8104803	Reverse	Y	0	342
GmIPT10	Glyma.13G209100.1	Chr13:32310692..32311377	Forward	N	0	154
GmIPT11	Glyma.11G188100.1	Chr11:25981523..25986063	Forward	Y	9	478
GmIPT12	Glyma.12G086300.1	Chr12:6987142..6990386	Reverse	Y	6	301
GmIPT13	Glyma.18G297300.1	Chr18:57479558..57480756	Forward	Y	0	333
GmIPT14	Glyma.08G364900.1	Chr08:47581368..47582279	Reverse	Y	1	293
GmIPT15	Glyma.17G045700.1	Chr17:3401249..3401828	Forward	N	0	75
GmIPT16	Glyma.13G271500.1	Chr13:37340399..37347149	Forward	Y	10	448
GmIPT17	Glyma.08G278400.1	Chr08:37592042..37592547	Reverse	N	1	69

*tRNA delta(2)-isopentenyl pyrophosphate transferase domain (IPP) predicted via Pfam.*

The BLAST searches were broadened to include the databases for common bean, *L. japonicus*, *M. truncatula* and the non-legume *Arabidopsis thaliana*. This confirmed the presence of 23 putative IPT genes in *M. truncatula*, making it the largest gene family compared with the eight, six and nine members identified in common bean, *L. japonicus* and *A. thaliana*, respectively (Takei et al., 2001; Azarakhsh et al., 2018) (**Supplementary Tables S3**–**S6**).

To further characterize the sequences obtained here, a multiple sequence alignment and phylogenetic tree were produced using Clustal Omega and PHYML. The multiple sequence alignments revealed a high level of conservation of amino acid residues among the different members of both the legume and non-legume *IPT* gene families (**Supplementary Figures S1**, **S2**). This is most evident between the amino acid residues situated at the positions 102–213 in the alignment at the N-terminal end of the protein sequences where the functional IPP domain is located.

**FIGURE 2:**
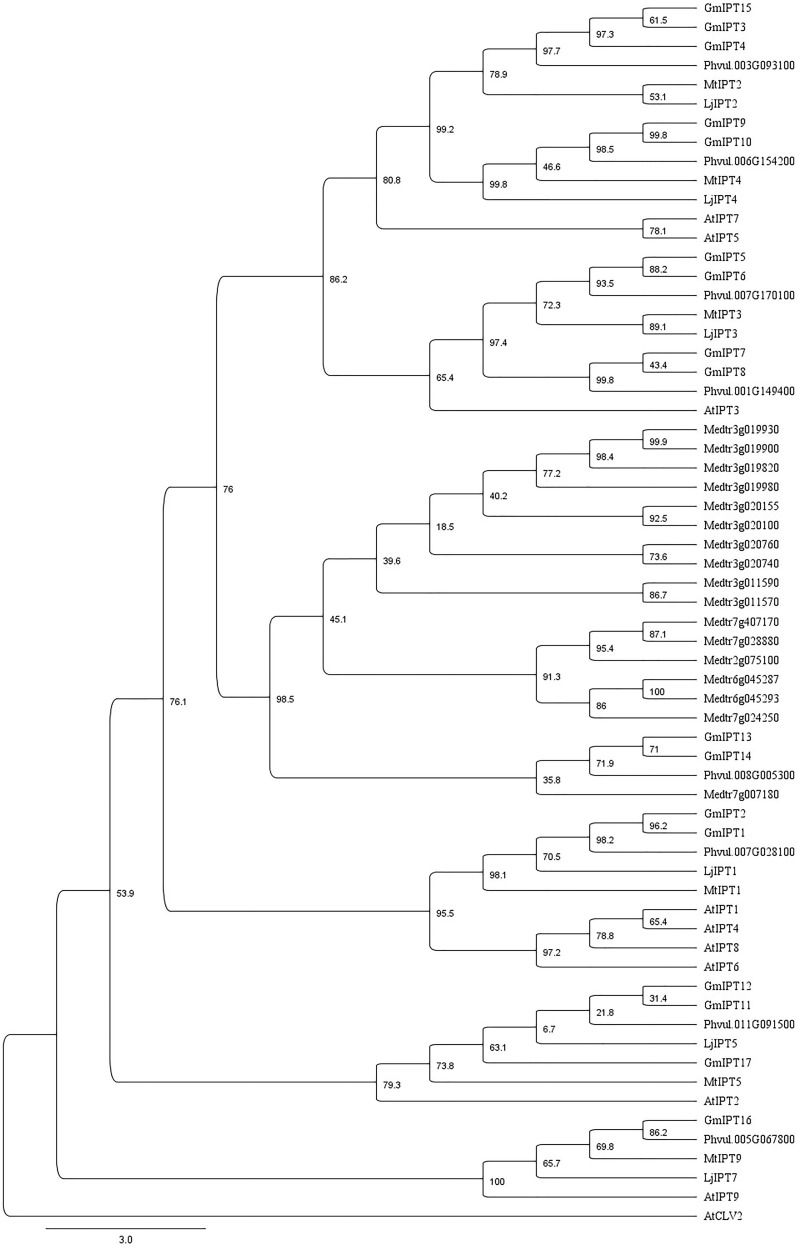
Characterization of IPT orthologs. Phylogenetic tree generated using a multiple sequence alignment of homologous amino acid sequences with 75% of the gap regions removed showing similarity between IPT orthologs of soybean, *L. japonicus*, *M. truncatula*, common bean (*P. vulgaris*) and *A. thaliana* including AtCLV2 as an outgroup. The phylogenetic tree is shown with bootstrap confidence values as percentages from 1,000 bootstrap replications.

Seven homeologous (i.e., duplicate) gene pairs are present within the soybean genome grouping together in the phylogenetic tree (**Figure 2**). This is a common occurrence with soybean genes due to previous whole genome duplication events 59 and 13 million years ago (Schmutz et al., 2010).

The orthologs of LjIPT3 are the homeologous duplicates, GmIPT5 and GmIPT6, with 75.1 and 72.2% amino acid sequence identity, respectively, while sharing 83.1% identity. Other soybean IPT family members clustering closely to LjIPT3, are the homeologous duplicates GmIPT7 and GmIPT8 with 50.8 and 49.8% amino acid identity. These four soybean genes are present in duplicated regions in the genome showing their close relationship. Homologous versions of LjIPT3 in common bean, *M. truncatula* and *A. thaliana* have the identifiers Phvul.007G170100, Medtr1g072540 (MtIPT3) and AT3G63110 (AtIPT3) with a protein sequence identity of 76, 74 and 53%, respectively.

**FIGURE 3:**
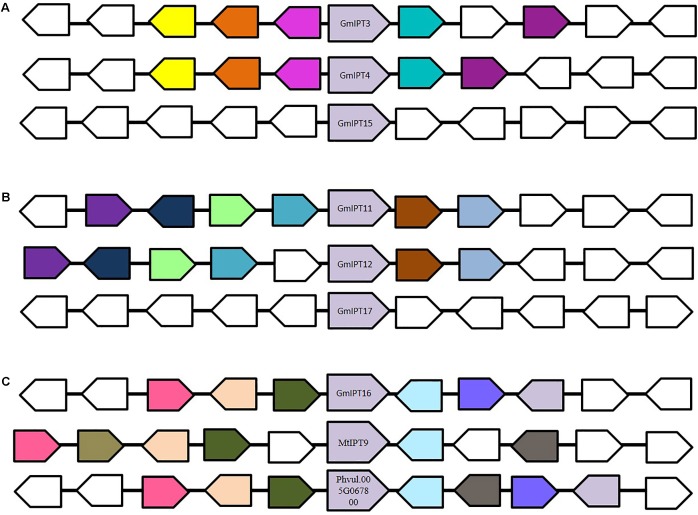
Genomic environment of **(A)** *GmIPT3/GmIPT4/GmIPT5*, **(B)** *GmIPT11/GmIPT12/GmIPT17* and **(C)** *GmIPT16* in soybean. The genes of interest are centrally positioned and highlighted in purple. Surrounding genes with an identical putative function are shown in the same colors with genes with an unrelated function left uncolored. The direction of the arrow indicates the gene direction compared to the *IPT* genes. **(A)** *GmIPT3* and *GmIPT4* are true homologous with a highly conserved genomic region. *GmPT15* arose as a tandem duplication of *GmPT3*, in which only the *IPT* gene was duplicated and not its environment. **(B)** *GmIPT11* and *GmIPT12* are homeologous duplicates represented by the high level of gene synteny. *GmIPT17* does not share this conservation. **(C)** *GmIPT16* does not share synteny with any of the other 16 soybean *IPT* genes, but its surrounding genes are highly identical to those surrounding its orthologs *MtIPT9* and *Phvul.005G067800* in *M. truncatula* and common bean (*P. vulgaris*).

Six *IPT* genes group into two groups of three genes (*GmIPT3/GmIPT4/GmIPT15* and *GmIPT11/GmIPT12/GmIPT17*) (**Figure 2**). The first group clusters closely to *LjIPT2* that was recently shown to be responsible for the initial cytokinin burst required for nodulation initiation (Reid et al., 2017). The genomic regions of *GmIPT3* and *GmIPT4* display a high level of synteny with five identical genes surrounding the two *IPT* genes (**Figure 3A**). This indicates that *GmIPT3* and *GmIPT4* are true homeologous duplicates. The third gene, *GmIPT15*, shares a protein sequence identity of 92.9% with *GmIPT3*. In addition, the two genes are present on the same chromosome (chromosome 17) and lack duplications of their genomic regions, indicating a recent tandem duplication of *GmIPT3* resulting in *GmIPT15*. Interestingly, the predicted GmIPT15 protein is truncated to only 75 amino acid residues and does not feature the highly conserved IPP amino acid sequence motif at the N-terminal end that is required for function. In addition to having a gap upstream of the predicted coding sequence that puts it out of frame, the currently available genome includes a 100 bp stretch of unconfirmed nucleic acid identity (denoted in the sequence as: -NNN-). Similar to GmIPT15, the GmIPT10 product is truncated with a stretch of nucleic acids of unconfirmed identity. This lack of identified nucleic acid residues in *GmIPT15* and *GmIPT10* may be due to the high level of sequence identity amongst the *IPT* genes and/or the presence of repetitive sequences within a gene, making the identity of some nucleic acid residues difficult to confirm with absolute certainty.

The second group (*GmIPT11/GmIPT12/GmIPT17*) is closely related to *LjIPT5* as well as *Phvul.011G091500* and *MtIPT5* in common bean and *M. truncatula*. The role of *LjIPT5* was previously investigated in nodule development and AON by Sasaki et al. (2014) and unlike *LjIPT3*, no differences in transcript levels were observed in the shoot after inoculation with compatible rhizobia. These orthologs are all characterized by the presence of a high number of introns (6–9 introns), except for *GmIPT17*, which has only one predicted intron (**Table 1**). A multiple sequence alignment of their genomic sequences using Clustal Omega indicated little conservation. *GmIPT11* and *GmIPT12* share 69.9% sequence identity and are located in a highly similar genomic region highlighted by the presence of six conserved genes (**Figure 3B**). The GmIPT12 protein is missing part of the N-terminal conserved region. Wrongly predicted intron locations might put the sequence out of frame leading to a truncated protein sequence. The genomic sequence of *GmIPT17* is much less conserved compared with *GmIPT11* and *GmIPT12* showing no conservation of the genomic region. *GmIPT17* likely does not have a homeologous duplicate. In addition, *GmIPT17* is predicted to be a truncated protein with only the central region translated. Like *GmIPT17*, *GmIPT16* is another gene that appears to completely lack a duplicate partner, indicating that it was lost or that *GmIPT16* arose after the most recent whole genome duplication event. A high level of genetic synteny is present for *GmIPT16* and orthologs in other legumes species including *MtIPT9* and *Phvul.005G067800* in contrast to *GmIPT17* (**Figure 3C**). Therefore, its homeologous duplicate was most likely lost over time.

### *IPT* Gene Expression in the Shoot and Root Following Rhizobia Inoculation

Previously, cytokinin was suggested to be the unidentified shoot-derived inhibitor in AON as demonstrated by the upregulation of *LjIPT3* in wild-type shoots, but not in LjHAR1 defective mutants (Sasaki et al., 2014). To identify *IPT* genes in soybean that are differentially regulated following rhizobia inoculation of the root, the expression of all 17 *IPT* genes was examined in both the root and shoot (i.e., trifoliate leaves) (**Supplementary Figure S3**). The first trifoliate leaf was tested as the GmNARK receptor is highly expressed in mature trifoliate leaves (Nontachaiyapoom et al., 2007). Tissues were harvested from wild-type Bragg and its supernodulating GmNARK mutant (*nts382*) inoculated with either compatible *B. diazoefficiens* USDA110 or its incompatible *nodC^−^* mutant strain as a negative control. GmNARK function is completely blocked in the *nts382* background, enabling characterization of possible IPT candidates involved in the AON pathway.

**FIGURE 4:**
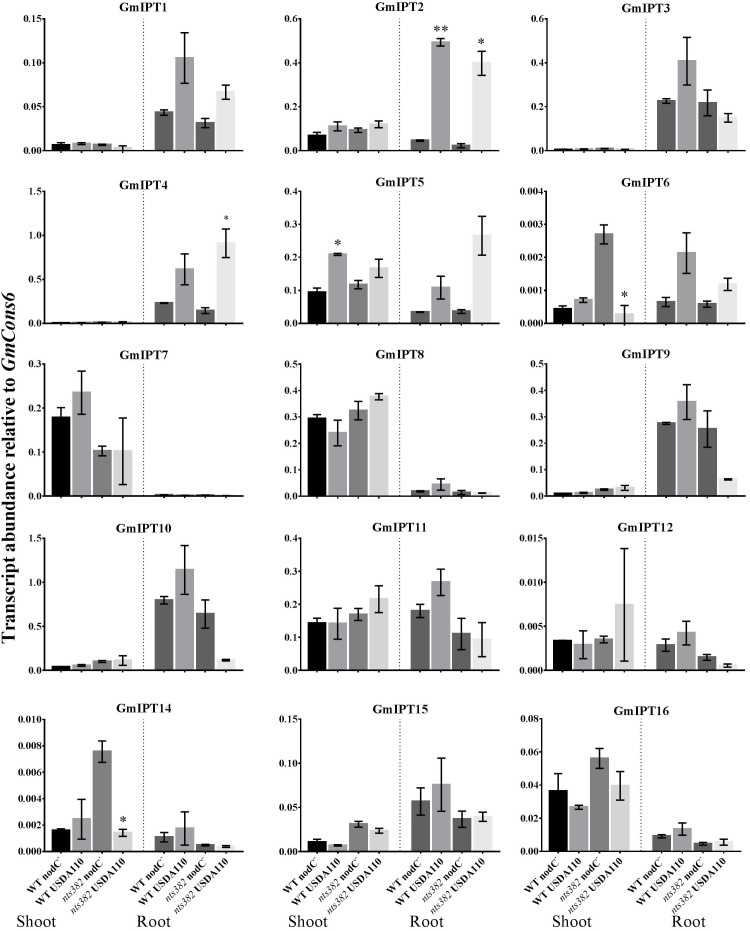
Expression of soybean *IPT* genes in the shoot and roots following inoculation with *B. diazoefficiens*. Two-week old wild-type Bragg (WT) and GmNARK mutant (*nts382*) plants were inoculated with *B. diazoefficiens* USDA110 or its isogenic and incompatible *nodC^−^* mutant. The first trifoliate leaf and total root were harvested 10 days after inoculation. Expression levels are relative to the housekeeping gene *GmCons6*. Bars represent mean ± SEM of two biological replicates (*n* = 6 plants per replicate). Asterisks show significant differences in expression (Student’s *t*-test, ^∗∗^*P* ≤ 0.01, ^∗^*P* ≤ 0.05).

The homeologous duplicates *GmIPT1* and *GmIPT2* respond to inoculation in the root, but not the shoot (**Figure 4**). This change in expression is significant for *GmIPT2* in both WT Bragg and GmNARK mutant (*nts382*) roots (*P* = 0.002 and *P* = 0.021, respectively); and is consistent with *LjIPT1* expression, the ortholog of *GmIPT1* and *GmIPT2*, which is upregulated in the root and not in the shoot (Chen et al., 2014; Sasaki et al., 2014).

Expression of the second homeologous pair, *GmIPT3* and *GmIPT4*, was root-specific and GmNARK-independent with transcript levels of *GmIPT4* increasing following inoculation (*P* = 0.161 in WT and *P* = 0.044 in *nts382*) (**Figure 4**). These genes cluster with *GmIPT15*, a duplicate of *GmIPT3* and possibly a pseudogene, which does not show a clear trend in expression in response to the presence of rhizobia. The ortholog of these genes is *LjIPT2*; it is expressed in roots in conjunction with *LjLOG4* and is reported to be required for the initial cytokinin burst in early nodulation (Reid et al., 2017).

**FIGURE 5:**
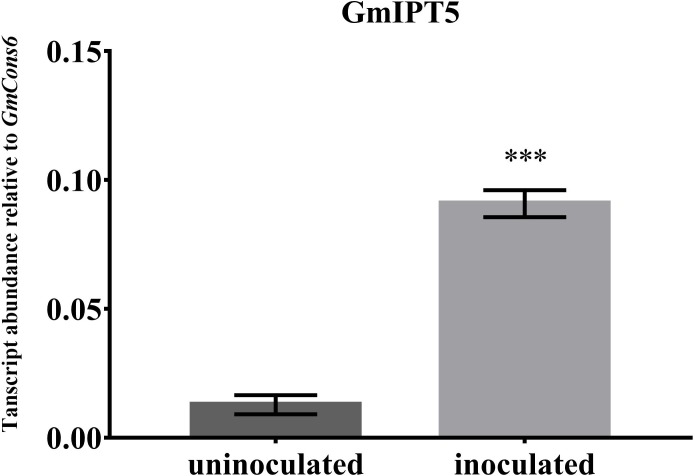
Expression of *GmIPT5* in the GmNARK mutant shoot following inoculation with *B. diazoefficiens*. Three-week old trifoliate leaves were harvested after plants were either inoculated with *B. diazoefficiens* or uninoculated. Expression levels are relative to the housekeeping gene *GmCons6*. Bars represent mean ± SEM of two biological replicates (*n* = 5 plants per replicate). Asterisk shows significant differences in expression (Student’s *t*-test, ^∗∗∗^*P* ≤ 0.001).

One of the orthologs of *LjIPT3*, *GmIPT5*, was found to be significantly induced in the wild-type shoot upon inoculation with compatible rhizobia (*P* = 0.014) (**Figure 4**). This trend of elevated *GmIPT5* expression was observed in the root as well (*P* = 0.165) and similarly observed in the GmNARK mutant root and shoot; however, these increases in expression were not significant (*P* = 0.060 and 0.244, respectively). As a trend of upregulation was noticeable, a second set of GmNARK mutant shoot samples was analyzed to determine whether *GmIPT5* expression is GmNARK-dependent or not (**Figure 5**). This established a significant increase in *GmIPT5* transcript levels in a GmNARK-independent manner (*P* = 0.0003). Expression of *GmIPT6*, the homeologous duplicate of *GmIPT5*, exhibited a similar pattern in response to inoculation with compatible rhizobia, with the exception of GmNARK mutant shoots, but the transcripts levels of *GmIPT6* were much lower compared to *GmIPT5* (*P* = 0.150 and *P* = 0.143 in shoot and root, respectively) and hence caution should be taken when assessing its expression here (**Supplementary Figure S3**). This difference in expression levels is often observed for soybean homeologous with one of the duplicates often becoming reduced or even silenced over long periods of time (Lynch and Conery, 2000; Granger et al., 2002). Likewise, a decrease in *GmIPT14* mRNA levels in the GmNARK mutant shoot was observed, but again the transcript levels were very low for this gene and may not be biologically relevant (**Figure 4** and **Supplementary Figure S3**). The homeologous partner of *GmIPT14*, *GmIPT13*, was omitted as its expression could not be detected.

*GmIPT7* and *GmIPT8* cluster closely to *GmIPT5*, *GmIPT6* and *LjIPT3* (**Figure 2**). They are shoot-specific in expression, but unlike *GmIPT5*, their expression remained constant after inoculation with compatible rhizobia in both wild-type and GmNARK mutant plants (**Figure 4**). *GmIPT9* and *GmIPT10* are root-specific in expression, but also were not differentially expressed by inoculation with the different rhizobia strains.

The remaining genes (*GmIPT11*, *GmIPT12* and *GmIPT16*) do not show a noticeable trend in shoot or root gene expression after inoculation with *B. diazoefficiens*. GmIPT17 was predicted to be truncated and its gene expression could not be detected. This gene is likely a pseudogene and is therefore not shown in **Figure 4**.

### *IPT* Gene Expression in the Root Following Nitrate Treatment

Nitrate-dependent regulation of nodulation acts locally in soybean as demonstrated by overexpression experiments using *GmNIC1a* where nodulation was only suppressed in transgenic, but not in non-transgenic roots on the same plant (Reid et al., 2011). Therefore, the expression of all 17 *IPT* genes of soybean was assessed in the root following nitrate-treatment to identify members of the gene family involved in nitrogen response and possibly nitrate-dependent regulation of nodulation.

**FIGURE 6:**
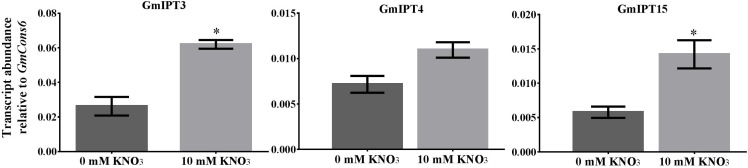
Expression of *GmIPT3*, *GmIPT4* and *GmIPT15* in response to nitrate. Soybean plants were treated with either 0 or 10 mM KNO_3_ and roots were harvested from 23-day-old plants. Expression levels are relative to the housekeeping gene *GmCons6*. Bars represent mean ± SEM of two biological replicates (*n* = 4 plants per replicate). Asterisk shows significant differences in expression (Student’s *t*-test, ^∗^*P* ≤ 0.05).

Nitrate treatment induced the upregulation of the duplicates *GmIPT3* (*P* = 0.027) and *GmIPT15* (*P* = 0.021) (**Figure 6**). The transcript level of the third copy clustering with these two genes, *GmIPT4*, also increased, but not significantly (*P* = 0.095). *GmIPT3* is expressed at levels twice that of both *GmIPT15* and *GmIPT4*, which showed a trend of upregulation in the rhizobia inoculated roots. Other rhizobia-responsive genes, e.g., *GmIPT2* and *GmIPT5*, as well as all remaining *IPT* genes, were not differentially regulated in the root in response to nitrate (**Supplementary Figure S4**).

## Discussion

Cytokinins are important regulators of plant growth and development, fine-tuning the balance between cell proliferation and differentiation. They are essential for rhizobia infection and nodule development through the initiation of cortical cell divisions and the induction of early nodulation transcription factors, such as NSP2 (Nodulation Signaling Pathway 2), NIN (Nodule Inception) and ERN1 (Ethylene-Responsive binding domain factor required for Nodulation 1), acting in an LHK1-dependent manner (Murray et al., 2007; Tirichine et al., 2007; Plet et al., 2011; van Zeijl et al., 2015). Indeed, a lack of cytokinin perception in the early stages of nodulation leads to infection thread formation without subsequent nodule organogenesis (Murray et al., 2007; Held et al., 2014). Here, we set out to further characterize the role of cytokinin and the *IPT* gene family in the AON and nitrate-regulation control pathways.

Exogenous application of cytokinin promoted nodule development at low concentrations, while high concentrations reduced nodulation. The former result supports a promoting effect of the hormone on nodulation, while the later finding is likely the result of toxicity induced by an excess level of the hormone (e.g., elevated ethylene production; Lorteau et al., 2001; Ferguson et al., 2005). This effect is particularly evident when BAP is directly applied to the root. BAP is a synthetic cytokinin that is very active and more stable in aqueous solution and once taken up by the plant than other cytokinin compounds (Deleuze et al., 1972). Despite the negative effect on plant growth and development, high doses of phytohormones are commonly used in current research and care should be taken in the selection of appropriate physiologically relevant concentrations in relation to the species and growing conditions used. The promoting effect was most evident when using petiole feeding to deliver the hormone directly and continuously into the plant mimicking the action of the shoot-derived inhibitor in AON, compared with root drenching, which is influenced by plant uptake, metabolism and the physical properties of the growing substrate. Exogenous cytokinin supply has previously been shown to induce nodulin genes, amyloplast depositions and the formation of nodule primordia (Bauer et al., 1996; Heckmann et al., 2011). Furthermore, overexpression of cytokinin biosynthesis genes and subsequent secretion by nodulation-defective rhizobia was reported to initiate organogenesis in alfalfa (Cooper and Long, 1994). Sasaki et al. (2014) also report an inhibitory effect on nodulation following the application of cytokinin (BAP) to the roots of *L. japonicus* and did not report a promotion of nodulation when using lower concentrations of cytokinin. However, these findings might not be contradictory and may instead reflect differences in cytokinin type and effective concentration range between species. Differences in age at harvest, growing conditions or another experimental parameter may also influence responses.

Cytokinin biosynthesis is catalyzed by IPT enzymes and an initial cytokinin burst is required to trigger nodule organogenesis (reviewed in Ferguson and Mathesius, 2014). Reid et al. (2017) recently demonstrated that LjIPT2, together with LjLOG4, is responsible for the build-up of cytokinins, independent to and upstream of the LHK1 cytokinin receptor in *L. japonicus*. In addition, *LjIPT3* is induced in the root after inoculation with compatible rhizobia (Chen et al., 2014; Sasaki et al., 2014). Sasaki et al. (2014) reported *LjIPT3* expression was also induced in the shoot and speculated that cytokinin might be the elusive shoot-derived inhibiting signal, acting downstream of the leucine-rich repeat receptor kinase LjHAR1 in AON.

In this study, 17 *IPT* genes were identified in the soybean genome. All members were found to have a homeologous duplicate or to be a duplicate of one of the homeologous copies, with the exception of *GmIPT16* and *GmIPT17*, which have no discernible partner. The comprehensive phylogenetic analysis reported here provides insight into the potential functions of these genes. The 17 family members of soybean are considerably more than the 8 and 6 members identified in common bean and *L. japonicus*. Having approximately double the number of gene family members of common bean is typical for soybean due to a whole genome duplication event that occurred roughly 13 million years ago (Schmutz et al., 2010; Hastwell et al., 2015a, 2017).

The orthologs of the root-induced *LjIPT2* (Reid et al., 2017) were identified as *GmIPT3* and *GmIPT4*, with *GmIPT15* identified as a duplicate of *GmIPT3*. The orthologs of the root- and shoot-induced *LjIPT3* (Chen et al., 2014; Sasaki et al., 2014) were found to be *GmIPT5* and its duplicate *GmIPT6*.

*IPT* gene expression was analyzed in both soybean shoots and roots of rhizobia-inoculated plants to identify potential candidates acting in nodulation and AON (Ferguson et al., 2018). *GmIPT5* was significantly induced in wild-type trifoliate leaves following inoculation of the root with compatible rhizobia. This is consistent with findings of Sasaki et al. (2014). Despite not being significant, the trend of increased *GmIPT5* transcript levels is also noticeable in the GmNARK mutant (*nts382*) in both the root and shoot. GmNARK defective plants that were either inoculated or non-inoculated confirmed a significant difference in *GmIPT5* transcript abundance. This seems to suggest that the upregulation of gene expression is independent of the GmNARK receptor and is unlikely to have a role in the AON pathway in soybean as suggested for *L. japonicus* by Sasaki et al. (2014) who found that regulation of *LjIPT3* is LjHAR1-dependent in the shoot. This might be due to any number of reasons including species-specific differences, different plant development stages, differences in growing conditions, etc. Expression of the homeologue of *GmIPT5*, *GmIPT6*, was considerably lower, indicating that it might not be biologically relevant in this process with *GmIPT5* being the dominant copy.

Upregulation of *LjIPT3* in the root has been reported to occur as early as 3 h after rhizobial inoculation (Chen et al., 2014) and as late as 10 days after inoculation (Reid et al., 2017). The latter finding is long after the CLE peptides, *LjCLE-RS1* and *LjCLE-RS2*, are induced to initiate the AON process (Okamoto et al., 2009). Furthermore, the differential regulation of nodulation-suppressing CLE peptides in *L. japonicus* and *M. truncatula* is dependent on the cytokinin-induced action of NIN, a transcription factor required for infection thread and nodule primordia formation (Schauser et al., 1999; Mortier et al., 2012; Soyano et al., 2014). Cytokinins and their *IPT* biosynthesis genes therefore seem to play a major role in the nodule development process upstream of AON rather than being a key factor in AON itself. Increased *GmIPT5* expression in the shoot after rhizobial inoculation might be the plant’s way to enhance shoot growth in preparation of an influx in nitrogen via nitrogen fixation, rather than cytokinins acting as the shoot-derived inhibitor in AON (Rahayu et al., 2005). In support of this is work by Chen et al. (2014) who show that RNA interference of LjIPT3 results in reduced shoot development and increased chlorophyll breakdown resulting in subsequent leaf senescence, even in the absence of rhizobia. AtIPT3-synthesised cytokinins induce the expression of shoot-type nitrate transporters (AtNRT) to translocate and partition nitrate in the shoot in the non-legume *A. thaliana* when plenty of nitrogen is available in the soil (Kiba et al., 2011).

Another gene, *GmIPT2*, was found to respond to rhizobial inoculation in the root in a clear GmNARK-independent manner. Its homeologous duplicate, *GmIPT1*, is root-specific as well and shows the same induction albeit less strong. This is consistent with the increased expression in the root but not the shoot of the ortholog of these genes in *L. japonicus*, *LjIPT1*, following rhizobia inoculation. The precise function of this gene remains unclear, but expression of *LjIPT1* is highest in the flower and only moderate in the roots (Chen et al., 2014).

The fourth gene responding to compatible rhizobia inoculation is the root-specific *GmIPT4*, the homeologue of *GmIPT3* that shows no clear change in expression. These genes are closely related to *LjIPT2*, which is required for the initial cytokinin burst during early nodulation events that are induced by rhizobia inoculation (Reid et al., 2017). Interestingly, expression of *GmIPT3* and its tandem duplicate *GmIPT15* (which is predicted to be truncated), is promoted in the root following nitrate treatment, but *GmIPT4* expression is not induced as strongly. Furthermore, transcript levels of *GmIPT4* are much higher than those of *GmIPT3* following rhizobia inoculation, while the opposite was observed in response to high soil nitrogen status. This might indicate that these genes have undergone genetic divergence (i.e., neofunctionalization) in which they developed a different function, as is sometimes observed with duplicated genes of soybean (Mirzaei et al., 2017).

In addition to the negative feedback system of AON, nodulation is controlled by environmental factors including the soil nitrogen status. In soybean, nitrate-dependent regulation of nodulation inhibits the nodulation process via a local pathway in contrast to systemic signaling required for AON (Hinson, 1975; Reid et al., 2011; reviewed in Ferguson et al., 2018). Therefore, only roots were tested in this study to investigate the role of cytokinins as the root-derived inhibitor in nitrate-dependent regulation of nodulation. In addition, *AtIPT* accumulation is highest in the root tissue in response to nitrogen, while expression in the shoot is relatively low (Takei et al., 2004). Systemic components of the nitrate-regulation of nodulation (e.g., nitrogen fixation, nodule size and nodule number) have been shown in other model legumes such as *M. truncatula* and *L. japonicus* (Jeudy et al., 2010; Soyano et al., 2014; Nishida et al., 2018; reviewed in Ferguson et al., 2018). Therefore, analysis of *IPT* gene expression in the shoot in response to soil nitrogen status could be investigated in future. The plant’s response to low nitrogen in the soil requires root phenotypic plasticity. This can occur through association with symbiotic soil rhizobia or through the proliferation of the lateral root system via a process called foraging. Similar to the establishment of nodule primordia, a precise cytokinin balance is essential for lateral root initiation and development. This process of foraging requires a reduction in cytokinin levels to increase lateral root proliferation. Overexpression of *IPT* genes in tobacco reduced root growth, while lateral root numbers are reduced in transgenic tobacco plants overexpressing a cytokinin oxidase (Hewelt et al., 1994; Werner et al., 2001). Here, the transcript levels of the cytokinin biosynthesis genes *GmIPT3* and *GmIPT15* are upregulated in the root in response to nitrogen. This is consistent with findings in *M. truncatula* showing the cytokinin receptor, MtCRE1, is required for both nodule and lateral root development, indicating mechanisms for nodule organogenesis have diverged from existing molecular mechanisms (Gonzalez-Rizzo et al., 2006). In addition, cytokinins are frequently found to act in an opposite manner during lateral root development and nodulation, requiring low or high cytokinin concentrations, respectively (Lohar et al., 2004). In *A. thaliana*, *AtIPT3* and *AtIPT5*, the homologous of *GmIPT3*, *GmIPT4* and *GmIPT15*, are upregulated in response to nitrate (Miyawaki et al., 2004; Takei et al., 2004). While *AtIPT3* is important in the short-term response to nitrate, *AtIPT5* is expressed after long-term exposure to different sources of nitrogen (Takei et al., 2004). Once plants encounter a high nitrogen patch, systemic nitrogen signaling is required to induce lateral root proliferation into the patch and inhibit lateral root development elsewhere. Several studies suggest a role in systemic nitrogen signaling for root-produced cytokinins (tZ) that move up the shoot where they regulate systemic nitrogen-pathways in response to a heterogeneous supply of soil nitrogen (Rahayu et al., 2005; Poitout et al., 2018). In addition, cytokinins in the root reduce nitrate uptake by inhibiting expression of high-affinity nitrate transporters (e.g., NRT2.1) when nitrogen availability is high, while shoot-type transporters are upregulated to translocate and redistribute nitrogen (Kiba et al., 2011). The results obtained here, together with the findings of Reid et al. (2017), suggest a general role for GmIPT3/GmIPT15 and their ortholog LjIPT2 in response to nitrogen status, possibly indicating that IPT-synthesized cytokinins act as regulators of root architecture and nitrate-dependent regulation of nodulation.

Collectively, our results established that low levels of cytokinins can promote nodulation whereas higher levels inhibit it. We also identified *IPT* genes that are regulated in either the root or shoot in response to inoculation with compatible rhizobia and additional *IPT* genes that are regulated in the root in response to nitrate. Our findings do not suggest that cytokinin is likely to be the shoot-derived inhibitor in AON. This is based on the upregulation of *GmIPT5*, an ortholog of *LjIPT3*, in a GmNARK-independent manner in the shoot combined with the nodulation promoting effect of low concentrations of cytokinin applied to the root and shoot. Indeed, using the petiole-feeding technique, the hormone was fed directly into the plant where SDI is produced and therefore would not be expected to promote nodule development. It should be noted that differences in species, treatment type, concentration range and cytokinin type might be important factors for differences in results reported here and in previous studies. While instead, *IPT* expression in the shoot possibly prepares the plant for new growth while awaiting a burst of nitrogen, which is often a limiting factor for plant growth. The comprehensive bioinformatic and expression analysis reported here can be used as tools to support future research into the role of cytokinin in nodule development. This could include establishing the exact function of various *IPT* genes in nodulation, including the role of increased *GmIPT5* expression in both shoot and root, in conjunction with the *LOG* genes that are crucial for cytokinin activation.

## Author Contributions

BF and PG devised the project and supervised. DL, CM and LH conducted the experiments. DL and CM performed bioinformatic and gene expression analyses. DL and LH performed petiole feeding and root drenching assays. CM analyzed the data, prepared the figures and wrote the manuscript with input from all authors. All authors read and approved the manuscript.

## Conflict of Interest Statement

The authors declare that the research was conducted in the absence of any commercial or financial relationships that could be construed as a potential conflict of interest.
