# Supplementary material for: Local and Systemic Effect of Cytokinins on Soybean Nodulation and Regulation of Their Isopentenyl Transferase (IPT) Biosynthesis Genes Following Rhizobia Inoculation
Source: Front Plant Sci. 2018 Aug 8;9:1150. doi: 10.3389/fpls.2018.01150 (PMC6092703; doi:10.3389/fpls.2018.01150)
Supplement: Supplementary file 1 [file Data_Sheet_1.PDF]

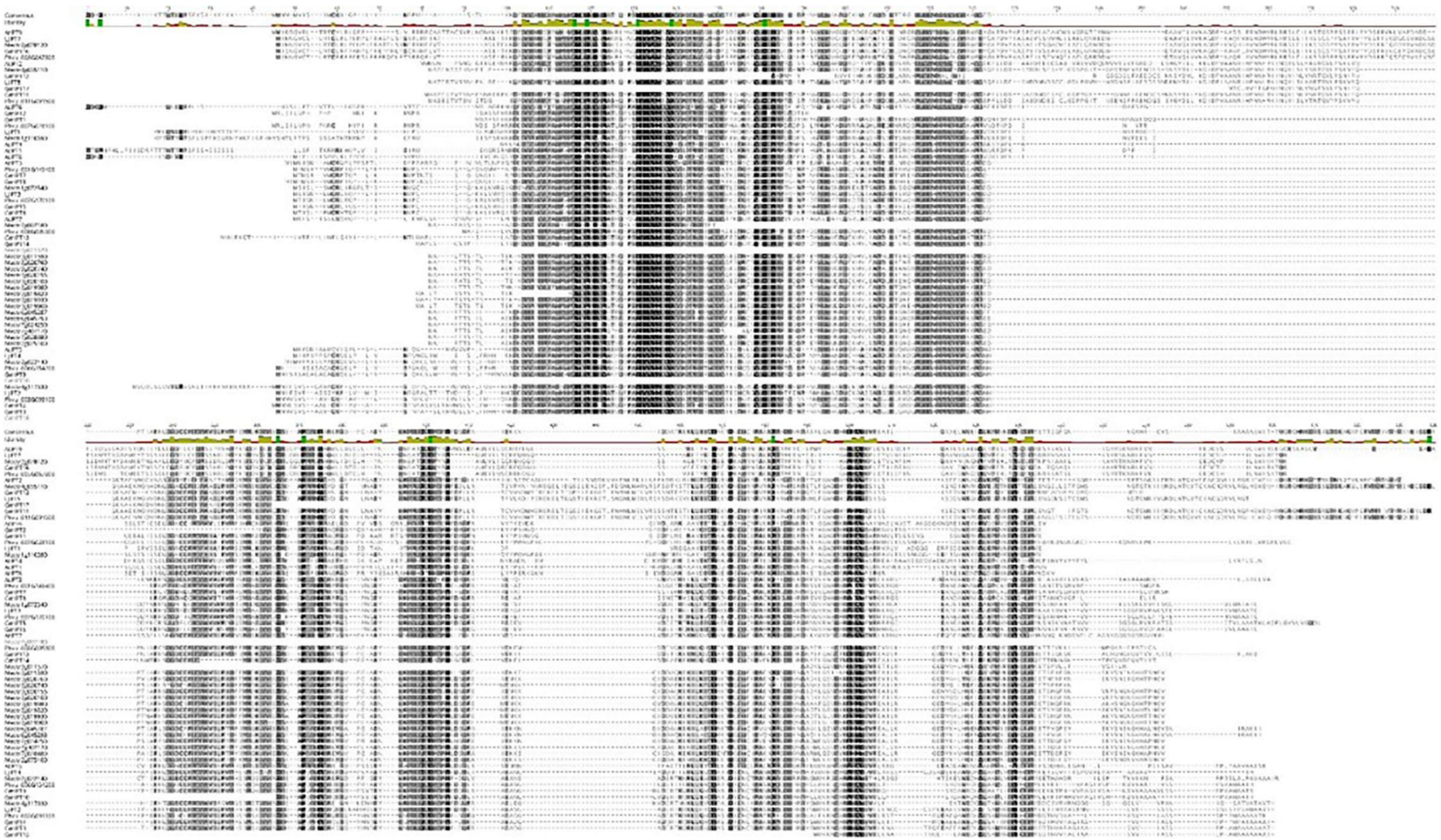

**Supplementary Figure S1. Multiple sequence alignment of IPT orthologues in soybean, *L. japonicus*, *M. truncatula*, common bean (*P. vulgaris*) and *A. thaliana*.** Shading represents conservation of amino acid residues at that position, with the darker the shading, the higher the conservation. The alignment was performed using Clustal Omega hosted by EMBL-EBI and visualised in Geneious Pro v6.8.1.

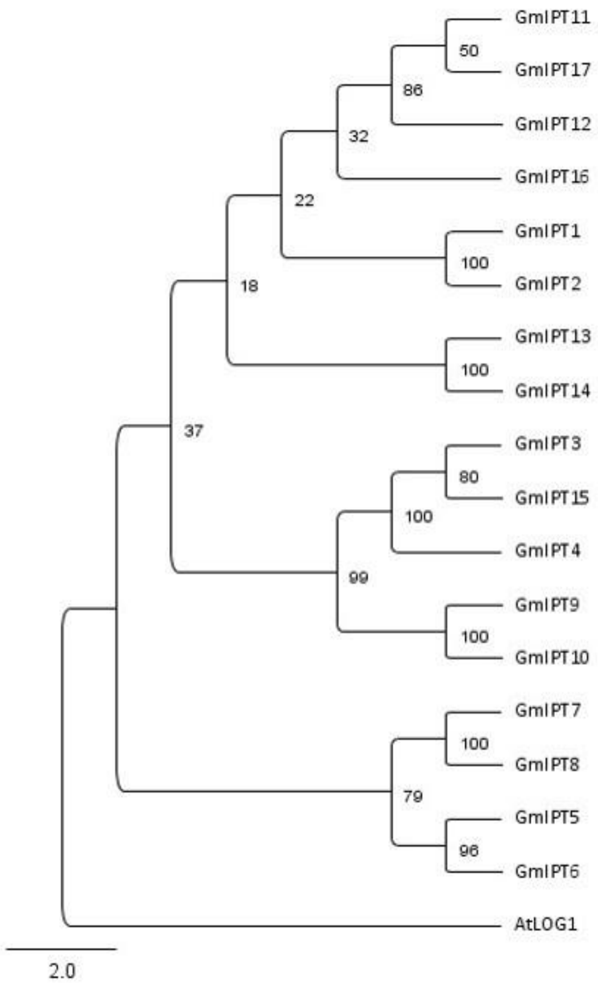


**Supplementary Figure S2. Characterisation of soybean IPT genes to identify homeologues (i.e. duplicate) copies.** The phylogenetic tree shows bootstrap confidence values as percentages from 100 bootstrap replications with AtLOG1 as and outgroup.

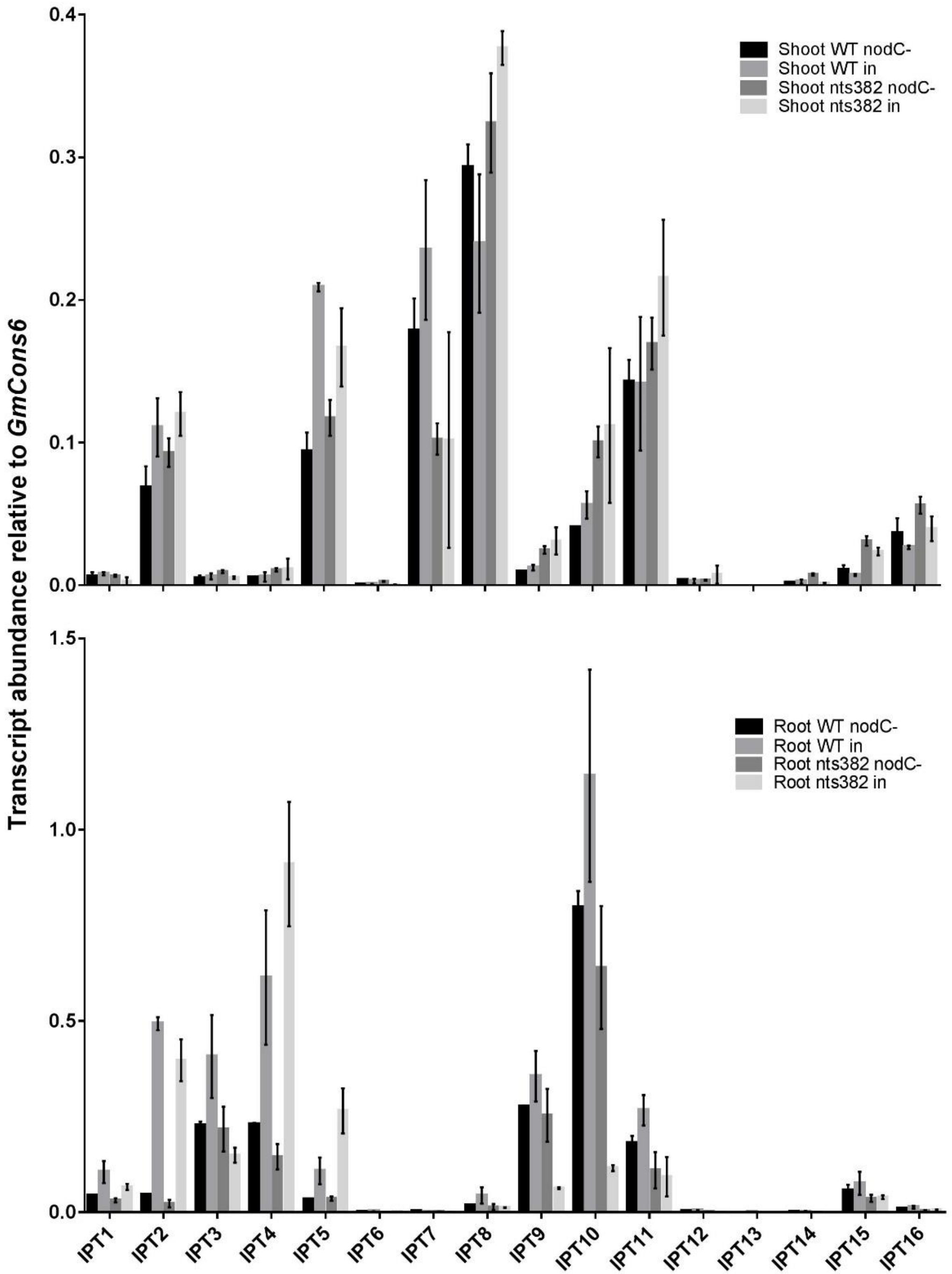


**Supplementary Figure S3. Expression of soybean *IPT* genes in the shoot and root in response to rhizobia inoculation.** Wild-type and GmNARK mutant (*nts382*) plants were inoculated at two weeks old with either *B. diazoefficiens* USDA110 (in) or its isogenic and incompatible *nodC*$^{-}$ mutant. Trifoliate leaves and roots were harvested ten days after inoculation. Expression levels are relative to the housekeeping gene *GmCons6*. Bars represent means ± SEM of two biological replicates (n = 6 plants per replicate). GmIPT17 expression was not detected and therefore excluded from this figure. (Note: this figure shows the same dataset presented in Figure 4. It allows for the comparison of relative transcript abundance between each of the *IPT* genes, but also highlights differences in expression between the shoot and root)

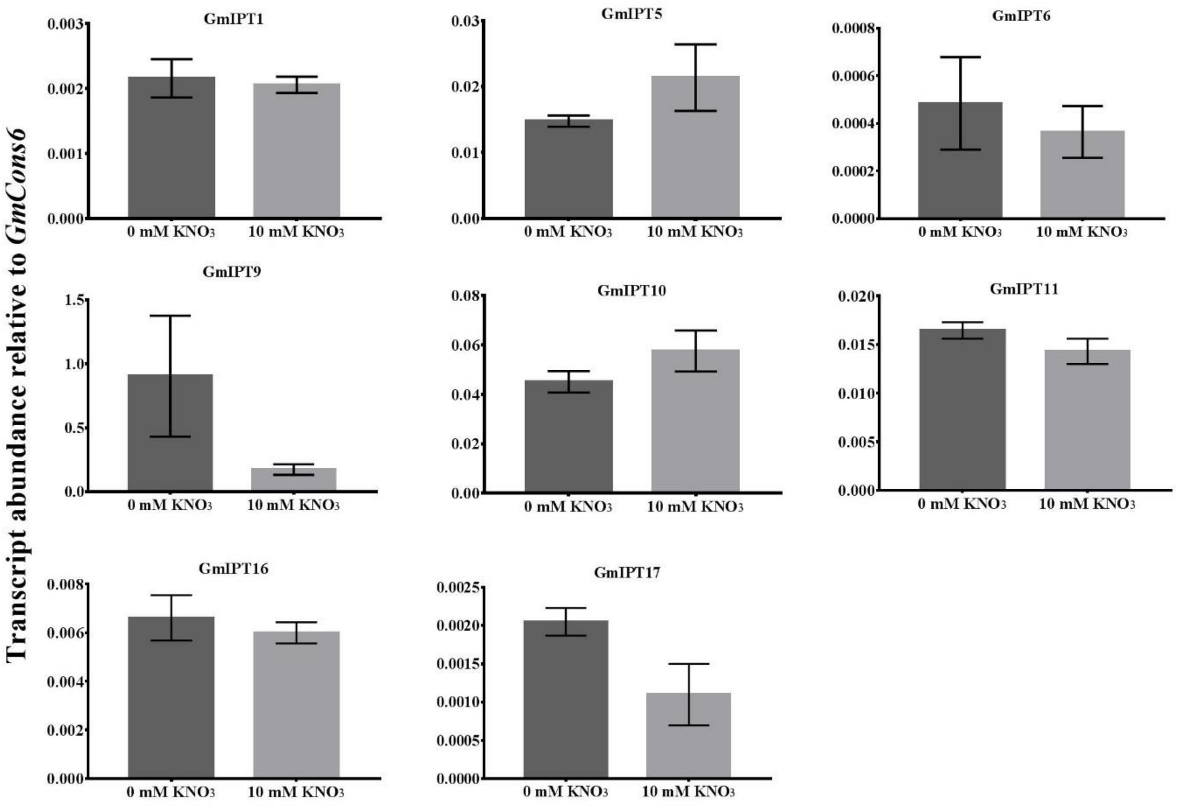


**Supplementary Figure S4. Expression of soybean *IPT* genes in response to nitrate.** Soybean plants were treated with either 0 or 10 mM $KNO_3$ and roots were harvested from 23 day-old plants. Expression levels are relative to the housekeeping gene *GmCons6*. Bars represent means ± SEM of two biological replicates (n = 4 plants). Genes for which expression was not detected using regular PCR are excluded from this figure.
