# Supplementary material for: Local and Systemic Effect of Cytokinins on Soybean Nodulation and Regulation of Their Isopentenyl Transferase (IPT) Biosynthesis Genes Following Rhizobia Inoculation
Source: Front Plant Sci. 2018 Aug 8;9:1150. doi: 10.3389/fpls.2018.01150 (PMC6092703; doi:10.3389/fpls.2018.01150)
Supplement: Supplementary file 2 [file Table_1.docx]

**Supplementary Table S1. List of primers used during this study for RT-qPCR analysis.**

| Gene | Reverse Primer | Forward Primer |
| --- | --- | --- |
| GmIPT1 | ATACACCGGACCCATTGCTC | CGGTGAAGGCGATTAAGGAT |
| GmIPT2 | GTGTTGTCCTTAATCGCCCG | GGTTCGGTCTCAGAAAGGCA |
| GmIPT3 | ATTGCGTGGCGAGGTCTATT | TGTCCCTTTTTCGTCACCAC |
| GmIPT4 | CATGCGTGTCCTCATACAGG | CGAGGTTTTCCTCAAGACCA |
| GmIPT5 | TCCTCGTCAACAACCTCTTCT | ATGGTGGATGAGCTGAGACC |
| GmIPT6 | TCAACCCAGAGGCAGAGAATA | TCAATCACGAGGTGCCAGAG |
| GmIPT7 | CATTCCGCCACCAATCATTT | CATACATGGAGGCCCTTGTG |
| GmIPT8 | CCCTTAACACTCCTCAACCTG | AACGAGACGAGGGTGAGTTT |
| GmIPT9 | TGATTGACCAACGCGTCTAA | ACTTCTGTCGCTACGCCACTT |
| GmIPT10 | ATGTTCCGTTTCCACAGCCC | AGAGGCTAATGGAGGCGTTG |
| GmIPT11 | CCCTCTGCTCTACATATCTGTCC | AGCAGAGAAGTGGGGTCAAGT |
| GmIPT12 | ACGGACACCAATGGCCTGA | GGATGCATCTCTCCGTGTACTT |
| GmIPT13 | TGAGGACATGCTTGCAAAAG | AAGGGCTTGGACATCATCAC |
| GmIPT14 | GTTTGAGCCTCCCACGATAA | TGTCGTGGTATTTCCCATCA |
| GmIPT15 | AATACATGCGGGAACGACAT | CCACCGAGGTTTTTCTCAAG |
| GmIPT16 | TCGAGAATGGTTTCCAGAGG | AAAGCATCACGGAATTTTGC |
| GmIPT17 | ACTTCTTTGCTGCCTTTCCT | GAATGCCTAACTGGGGATGA |

**Supplementary Table S2. Similarity between IPT amino acid sequences in soybean (*Glycine max*).** Values represent percentage identity. Note: the identity of some base pairs in the nucleotide sequence of *GmIPT10* and *GmIPT15* are not confirmed in the current version of the soybean genome (Phytozome Wm82.a2.v1).

|  | **IPT1** | **IPT2** | **IPT3** | **IPT4** | **IPT5** | **IPT6** | **IPT7** | **IPT8** | **IPT9** | **IPT10** | **IPT11** | **IPT12** | **IPT13** | **IPT14** | **IPT15** | **IPT16** | **IPT17** |
| --- | --- | --- | --- | --- | --- | --- | --- | --- | --- | --- | --- | --- | --- | --- | --- | --- | --- |
| **IPT1** |  | 91.90 | 48.38 | 48.16 | 52.21 | 50.92 | 48.70 | 49.81 | 48.71 | 42.50 | 41.58 | 35.50 | 46.89 | 42.48 | 37.21 | 28.57 | 44.19 |
| **IPT2** |  |  | 46.12 | 46.30 | 51.89 | 49.76 | 47.89 | 50.23 | 45.83 | 40.68 | 41.23 | 34.62 | 45.79 | 42.72 | 38.10 | 28.64 | 66.67 |
| **IPT3** |  |  |  | 89.49 | 47.81 | 48.41 | 50.83 | 49.83 | 67.89 | 69.01 | 38.11 | 32.65 | 41.51 | 41.07 | 94.67 | 24.17 | 48.39 |
| **IPT4** |  |  |  |  | 46.54 | 47.44 | 52.19 | 51.34 | 68.21 | 68.09 | 38.03 | 32.31 | 41.72 | 41.24 | 84.72 | 25.59 | 51.61 |
| **IPT5** |  |  |  |  |  | 83.18 | 62.01 | 62.62 | 44.69 | 40.27 | 35.71 | 32.00 | 45.08 | 42.01 | 36.23 | 28.05 | 35.48 |
| **IPT6** |  |  |  |  |  |  | 62.54 | 63.16 | 46.08 | 42.18 | 36.48 | 31.31 | 46.33 | 43.36 | 34.38 | 28.57 | 38.71 |
| **IPT7** |  |  |  |  |  |  |  | 87.91 | 46.69 | 38.97 | 40.69 | 31.82 | 41.33 | 39.36 | 33.96 | 25.87 | 48.28 |
| **IPT8** |  |  |  |  |  |  |  |  | 46.84 | 40.15 | 41.61 | 33.16 | 41.95 | 40.29 | 30.00 | 25.44 | 48.28 |
| **IPT9** |  |  |  |  |  |  |  |  |  | 92.81 | 36.83 | 32.00 | 40.94 | 40.78 | 60.94 | 23.84 | 48.39 |
| **IPT10** |  |  |  |  |  |  |  |  |  |  | 28.86 | 30.15 | 32.88 | 30.71 | 64.41 | 14.63 | 0.00 |
| **IPT11** |  |  |  |  |  |  |  |  |  |  |  | 81.88 | 34.98 | 33.33 | 16.42 | 22.11 | 79.71 |
| **IPT12** |  |  |  |  |  |  |  |  |  |  |  |  | 31.50 | 29.47 | 20.41 | 16.47 | 71.64 |
| **IPT13** |  |  |  |  |  |  |  |  |  |  |  |  |  | 80.90 | 23.81 | 22.97 | 51.61 |
| **IPT14** |  |  |  |  |  |  |  |  |  |  |  |  |  |  | 23.64 | 24.44 | 47.62 |
| **IPT15** |  |  |  |  |  |  |  |  |  |  |  |  |  |  |  | 6.67 | 0.00 |
| **IPT16** |  |  |  |  |  |  |  |  |  |  |  |  |  |  |  |  | 10.45 |
| **IPT17** |  |  |  |  |  |  |  |  |  |  |  |  |  |  |  |  |  |

**Supplementary Table S3. Similarity between IPT amino acid sequences in common bean (*Phaseolus vulgaris*).** Values represent percentage identity.

|  | **1** | **2** | **3** | **4** | **5** | **6** | **7** | **8** |
| --- | --- | --- | --- | --- | --- | --- | --- | --- |
| 1. **Phvul.007G028100** |  | 42.45 | 44.97 | 44.85 | 43.44 | 38.58 | 38.70 | 26.01 |
| 1. **Phvul.003G093100** |  |  | 47.30 | 47.16 | 67.71 | 36.51 | 43.60 | 25.25 |
| 1. **Phvul.007G170100** |  |  |  | 59.67 | 47.13 | 36.63 | 45.14 | 26.26 |
| 1. **Phvul.001G149400** |  |  |  |  | 46.98 | 41.96 | 44.16 | 23.79 |
| 1. **Phvul.006G154200** |  |  |  |  |  | 41.78 | 45.26 | 23.91 |
| 1. **Phvul.011G091500** |  |  |  |  |  |  | 33.45 | 24.12 |
| 1. **Phvul.008G005300** |  |  |  |  |  |  |  | 25.74 |
| 1. **Phvul.005G067800** |  |  |  |  |  |  |  |  |

**Supplementary Table S4. Similarity between IPT amino acid sequences in *Medicago truncatula*.** Values represent percentage identity.

|  | **2** | **3** | **4** | **5** | **6** | **7** | **8** | **9** | **10** | **11** | **12** | **13** | **14** | **15** | **16** | **17** | **18** | **19** | **20** | **21** | **22** | **23** |
| --- | --- | --- | --- | --- | --- | --- | --- | --- | --- | --- | --- | --- | --- | --- | --- | --- | --- | --- | --- | --- | --- | --- |
| 1. **Medtr1g110590** (MtIPT1) | 40.95 | 46.92 | 42.28 | 40.33 | 43.86 | 25.53 | 45.16 | 45.16 | 45.91 | 45.91 | 45.91 | 45.20 | 45.13 | 46.98 | 45.00 | 43.70 | 45.74 | 45.39 | 45.36 | 45.71 | 45.52 | 39.58 |
| 1. **Medtr4g117330** (MtIPT2) |  | 46.52 | 62.39 | 40.07 | 47.37 | 25.08 | 42.86 | 42.86 | 42.57 | 42.57 | 44.64 | 43.94 | 43.16 | 44.64 | 42.36 | 41.73 | 42.76 | 42.41 | 45.14 | 45.52 | 43.90 | 21.15 |
| 1. **Medtr1g072540** (MtIPT3) |  |  | 43.79 | 39.26 | 42.11 | 25.82 | 45.45 | 45.45 | 43.92 | 43.92 | 43.85 | 46.18 | 44.37 | 44.10 | 45.99 | 45.13 | 44.98 | 44.64 | 46.34 | 45.94 | 45.80 | 24.56 |
| 1. **Medtr2g022140** (MtIPT4) |  |  |  | 38.00 | 44.64 | 25.18 | 42.27 | 42.27 | 41.20 | 41.20 | 45.05 | 44.71 | 43.60 | 45.73 | 41.44 | 40.43 | 41.84 | 41.50 | 4384 | 43.42 | 43.64 | 12.50 |
| 1. **Medtr4g055110** (MtIPT5) |  |  |  |  | 33.93 | 23.73 | 36.90 | 36.90 | 36.73 | 36.73 | 36.99 | 34.93 | 36.11 | 36.64 | 35.74 | 35.48 | 37.46 | 37.11 | 37.11 | 35.23 | 36.21 | 22.81 |
| 1. **Medtr7g007180** |  |  |  |  |  | 32.14 | 60.71 | 60.71 | 56.14 | 56.14 | 61.40 | 57.89 | 59.65 | 63.16 | 59.65 | 48.89 | 52.63 | 52.63 | 52.63 | 61.40 | 59.65 | 0.00 |
| 1. **Medtr2g078120** (MtIPT9) |  |  |  |  |  |  | 25.98 | 25.98 | 25.00 | 25.00 | 25.39 | 27.34 | 26.19 | 25.78 | 25.49 | 23.77 | 24.63 | 24.61 | 24.61 | 27.24 | 25.88 | 8.82 |
| 1. **Medtr3g020155** |  |  |  |  |  |  |  | 100 | 76.16 | 76.16 | 80.46 | 79.80 | 78.52 | 79.14 | 88.74 | 87.59 | 89.07 | 88.74 | 88.74 | 88.50 | 86.71 | 67.74 |
| 1. **Medtr3g020100** |  |  |  |  |  |  |  |  | 76.16 | 76.16 | 80.46 | 79.80 | 78.52 | 79.14 | 88.74 | 87.59 | 89.07 | 88.74 | 88.74 | 88.50 | 86.71 | 67.74 |
| 1. **Medtr6g045287** |  |  |  |  |  |  |  |  |  | 100 | 86.18 | 84.54 | 82.67 | 83.88 | 74.59 | 73.88 | 73.27 | 72.94 | 76.57 | 76.39 | 76.16 | 54.84 |
| 1. **Medtr6g045293** |  |  |  |  |  |  |  |  |  |  | 86.18 | 84.54 | 82.67 | 83.88 | 74.59 | 73.88 | 73.27 | 72.94 | 76.57 | 76.39 | 76.16 | 54.84 |
| 1. **Medtr7g024250** |  |  |  |  |  |  |  |  |  |  |  | 87.83 | 86.67 | 88.16 | 77.56 | 76.63 | 77.23 | 76.90 | 80.20 | 80.21 | 79.47 | 58.06 |
| 1. **Medtr2g075100** |  |  |  |  |  |  |  |  |  |  |  |  | 89.67 | 90.46 | 77.23 | 76.63 | 76.90 | 76.57 | 80.20 | 78.47 | 79.47 | 56.45 |
| 1. **Medtr7g407170** |  |  |  |  |  |  |  |  |  |  |  |  |  | 91.33 | 76.25 | 75.26 | 75.25 | 74.92 | 78.93 | 76.06 | 77.85 | 61.29 |
| 1. **Medtr7g028880** |  |  |  |  |  |  |  |  |  |  |  |  |  |  | 76.90 | 76.63 | 76.57 | 76.24 | 79.87 | 77.78 | 80.13 | 56.45 |
| 1. **Medtr3g019980** |  |  |  |  |  |  |  |  |  |  |  |  |  |  |  | 90.03 | 89.44 | 89.11 | 86.47 | 87.50 | 85.43 | 69.35 |
| 1. **Medtr3g019820** |  |  |  |  |  |  |  |  |  |  |  |  |  |  |  |  | 94.54 | 94.2 | 86.60 | 85.14 | 84.48 | 64.52 |
| 1. **Medtr3g019930** |  |  |  |  |  |  |  |  |  |  |  |  |  |  |  |  |  | 99.67 | 87.13 | 86.81 | 85.43 | 70.97 |
| 1. **Medtr3g019900** |  |  |  |  |  |  |  |  |  |  |  |  |  |  |  |  |  |  | 86.80 | 86.46 | 85.10 | 70.97 |
| 1. **Medtr3g020760** |  |  |  |  |  |  |  |  |  |  |  |  |  |  |  |  |  |  |  | 91.67 | 88.41 | 70.97 |
| 1. **Medtr3g020740** |  |  |  |  |  |  |  |  |  |  |  |  |  |  |  |  |  |  |  |  | 91.67 | 80.36 |
| 1. **Medtr3g011590** |  |  |  |  |  |  |  |  |  |  |  |  |  |  |  |  |  |  |  |  |  | 77.05 |
| 1. **Medtr3g011570** |  |  |  |  |  |  |  |  |  |  |  |  |  |  |  |  |  |  |  |  |  |  |

**Supplementary Table S5. Similarity between IPT amino acid sequences in *Lotus japonicus*.** Values represent percentage identity, corresponding Lotus Base identifiers are shown.

|  | **LjIPT1** | **LjIPT2** | **LjIPT3** | **LjIPT4** | **LjIPT5** | **LjIPT7** |
| --- | --- | --- | --- | --- | --- | --- |
| **LjIPT1**  Lj5g3v2183860.1 |  | 39.51 | 42.86 | 39.38 | 27.90 | 17.22 |
| **LjIPT2**  Lj4g3v2400530.1 |  |  | 47.09 | 62.10 | 27.13 | 14.73 |
| **LjIPT3**  Lj5g3v0962690.1 |  |  |  | 47.45 | 28.31 | 15.84 |
| **LjIPT4**  Lj0g3v0154359.1 |  |  |  |  | 25.83 | 15.47 |
| **LjIPT5**  Lj3g3v2996020.2 |  |  |  |  |  | 15.45 |
| **LjIPT7**  Lj3g3v1063700.1 |  |  |  |  |  |  |

**Supplementary Table S6. Similarity between IPT amino acid sequences in *Arabidopsis thaliana*.** Values represent percentage identity.

|  | **AtIPT1** | **AtIPT2** | **AtIPT3** | **AtIPT4** | **AtIPT5** | **AtIPT6** | **AtIPT7** | **AtIPT8** | **AtIPT9** |
| --- | --- | --- | --- | --- | --- | --- | --- | --- | --- |
| **AtIPT1**  AT1G68460.1 |  | 35.02 | 44.92 | 56.61 | 42.91 | 52.25 | 42.76 | 58.64 | 26.30 |
| **AtIPT2**  AT2G27760.1 |  |  | 33.11 | 35.74 | 39.38 | 33.78 | 36.68 | 32.78 | 24.74 |
| **AtIPT3**  AT3G63110.1 |  |  |  | 43.16 | 53.89 | 41.39 | 46.73 | 46.46 | 25.34 |
| **AtIPT4**  AT4G24650.1 |  |  |  |  | 38.41 | 46.82 | 40.48 | 55.56 | 23.36 |
| **AtIPT5**  AT5G19040.1 |  |  |  |  |  | 38.44 | 53.37 | 42.07 | 25.43 |
| **AtIPT6**  AT1G25410.1 |  |  |  |  |  |  | 38.31 | 52.81 | 22.65 |
| **AtIPT7**  AT3G23630.1 |  |  |  |  |  |  |  | 41.38 | 23.88 |
| **AtIPT8**  AT3G19160.1 |  |  |  |  |  |  |  |  | 23.79 |
| **AtIPT9**  AT5G20040.3 |  |  |  |  |  |  |  |  |  |
